# Kinetic Characterization of Novel HIV-1 Entry Inhibitors: Discovery of a Relationship between Off-Rate and Potency

**DOI:** 10.3390/molecules23081940

**Published:** 2018-08-03

**Authors:** Megan E. Meuser, Michael B. Murphy, Adel A. Rashad, Simon Cocklin

**Affiliations:** 1 Department of Biochemistry & Molecular Biology, Drexel University College of Medicine, Rooms 10307, 10309, and 10315, 245 North 15th Street, Philadelphia, PA 19102, USA; mem484@drexel.edu (M.E.M.); aaa396@drexel.edu (A.A.R.); 2 GE Healthcare Life Sciences, 100 Results Way, Marlborough, MA 01752, USA; Michael.B.Murphy@ge.com

**Keywords:** bioisosteres, HIV-1 Env, antiviral, surface plasmon resonance, computer-aided drug design

## Abstract

The entry of HIV-1 into permissible cells remains an extremely attractive and underexploited therapeutic intervention point. We have previously demonstrated the ability to extend the chemotypes available for optimization in the entry inhibitor class using computational means. Here, we continue this effort, designing and testing three novel compounds with the ability to inhibit HIV-1 entry. We demonstrate that alteration of the core moiety of these entry inhibitors directly influences the potency of the compounds, despite common proximal and distal groups. Moreover, by establishing for the first time a surface plasmon resonance (SPR)-based interaction assay with soluble recombinant SOSIP Env trimers, we demonstrate that the off-rate (k_d_) parameter shows the strongest correlation with potency in an antiviral assay. Finally, we establish an underappreciated relationship between the potency of a ligand and its degree of electrostatic complementarity (EC) with its target, the Env complex. These findings not only broaden the chemical space in this inhibitor class, but also establish a rapid and simple assay to evaluate future HIV-1 entry inhibitors.

## Highlights:

- The HIV-1 entry process represents an extremely attractive but underexploited therapeutic intervention point.
- Novel compounds with HIV-1 entry inhibitory activity were designed via field-based computational methods, synthesized and characterized biologically.
- A surface plasmon resonance (SPR) interaction assay was established for the first time for this class of antivirals, allowing assessment of affinity and kinetic parameters.
- The potency of a given entry inhibitor could be correlated with its dissociation rate parameter (k_d_) and its degree of electrostatic complementarity (EC) with its target, the Env complex.

## 1. Introduction

Because of the emergence of drug-resistant strains and the cumulative toxicities associated with current therapies, demand remains for new inhibitors of HIV-1 replication. An attractive intervention point for such new inhibitors is viral entry into permissive cells. Only two entry inhibitors are used in the clinic, enfuvirtide (Fuzeon) and maraviroc (Selzentry), each with their own limitations. Enfuvirtide’s clinical application is limited by the fact that it is a peptide, which is not orally bioavailable and therefore has to be injected, sometimes causing serious local injection reactions. Moreover, it requires twice-daily dosage at 90 mg per dose, leading to high cost that is not feasible for treatment in developing countries. Maraviroc, although well tolerated, is only active against R5-tropic viruses as it blocks HIV-1 entry via targeting the host cell coreceptor CCR5. Ideally, one would hope to be able to develop virally-targeted, small-molecule HIV-1 entry inhibitors that are orally bioavailable.

Entry of HIV-1 into susceptible cells is orchestrated by the Env complex present on the exterior of the virion. Env is composed of a heterotrimer of gp41 and gp120 subunits that undergo a series of interactions and conformational changes that result in the fusion of the viral and cellular membranes. Over recent years, a great deal has been learnt about the structure and thermodynamics of this process and which points in this cascade are susceptible to inhibition.

In the HIV-1 entry field, two main small-molecule inhibitor chemotypes, which target the Env complex, predominate: the NBD-556 analogs [1] and the BMS-378806 analogs [2]. The NBD-556 analogs are at a very early stage of development, whereas the BMS-378806-type compounds have been entered into clinical trials a number of times. Although potent across many of the major subtypes of HIV-1, the piperazine-based BMS-378806 analogs suffer from poor pharmacokinetic properties (primarily low bioavailability); deficiencies that Bristol-Myers Squibb tried to address using a prodrug approach. BMS-663068, a phosphonooxymethyl prodrug of BMS-626529 [3], the current lead compound being developed by ViiV Healthcare under the name fostemsavir, is currently in phase III clinical trials. Despite encouraging results, fostemsavir is only recommended for highly treatment-experienced patients and requires dosages of up to 600 mg twice daily.

Our group has been exploring the computationally-driven modification of the BMS-type entry inhibitors in an overarching effort to bring this class of inhibitors out of the realm of salvage therapy [4,5]. In this study, we demonstrate the continued utility of our computational approach for modification of inhibitory compounds and subsequent modulation of their drug-like properties. However, most importantly, we demonstrate a strong correlation between the off-rate values of the compounds for a soluble recombinant trimer, obtained by a novel surface plasmon resonance (SPR) interaction assay, and their potency in a single-round infection assay. This new information, along with the novel SPR assay, will significantly increase the throughput, assessment, and prioritization of future analogs within our research program.

## 2. Results and Discussion

### 2.1. Design of Compounds ***SC16***, ***SC18***, and ***SC39***

**Figure 1:**
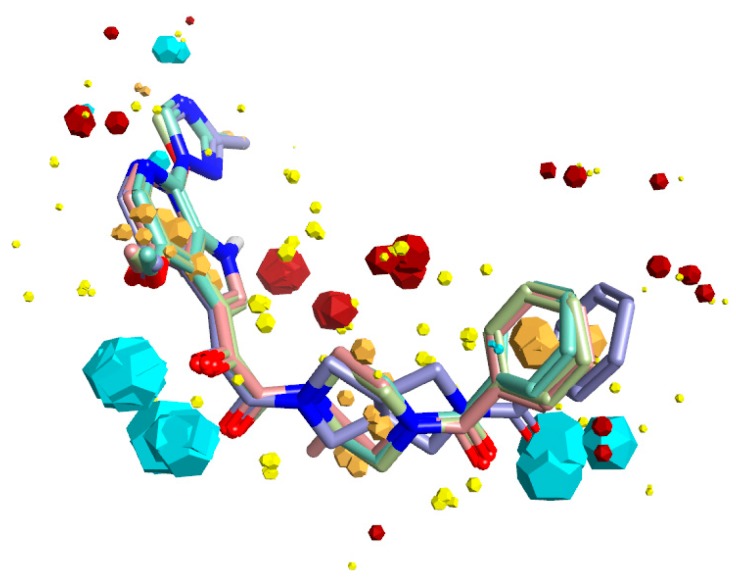
Bioactive conformation hypothesis and field-point pharmacophore of BMS-377806, BMS-488043, BMS-626529, and SC11. Blue field points = negatively charged; red = positively charged. Yellow spheres = van der Waals; gold = hydrophobic. Oxygen atoms are shown in red, nitrogen in blue. The size of the field point is directly related to the strength of the interaction.

**Scheme 1:**
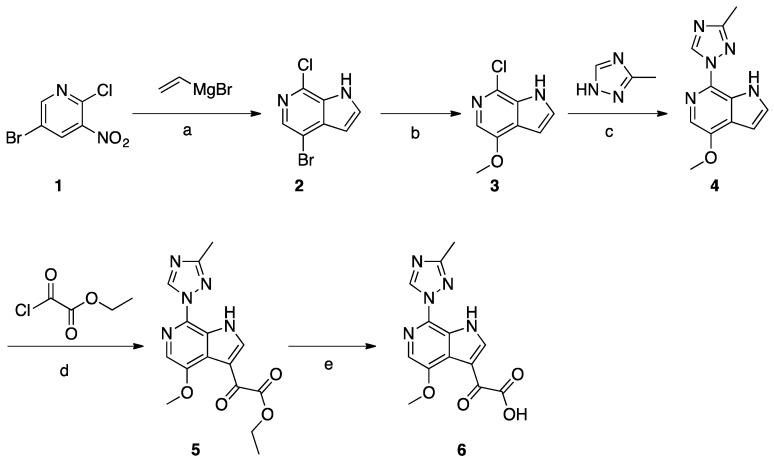
Reagents and conditions: (a) vinylmagnesium bromide, THF, −78 °C to −40 °C; (b) NaOMe, CuI, MeOH, microwave, 110 °C; (c) neat copper powder, KOH, N_2_ atmosphere, 170–175 °C; (d) ethylmagnesium bromide, ethyl-2-chloro-2-oxoacetate, pyridine, THF, −45 °C to −10 °C; (e) NaOH, MeOH, H_2_O.

**Scheme 2:**
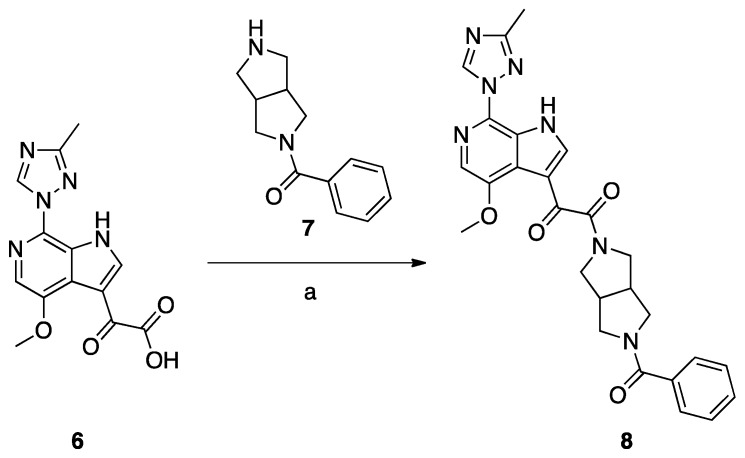
Reagents and conditions: (a) DEPBT, DIEA, DMF room temperature, 12 h.

**Scheme 3:**
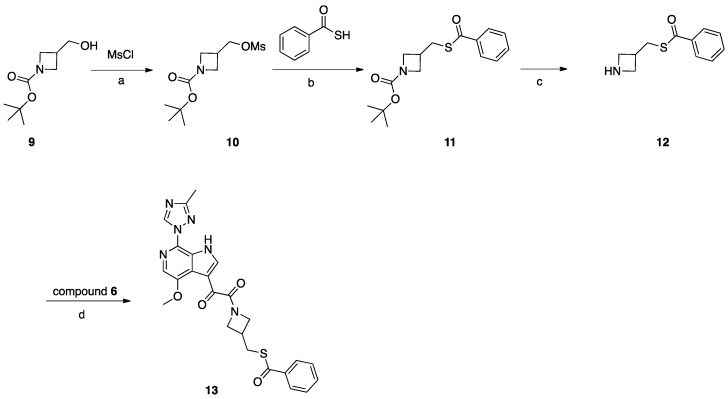
Reagents and conditions: (a) MsCl, NMM, DCM, 0–25 °C; (b) benzothioic S-acid, K_2_CO_3_, DMF, 80 °C, 2 h; (c) TFA, DCM, 0–25 °C, 3 h; (d) HATU, DIEA, DMF, 20 °C, 18 h.

**Scheme 4:**
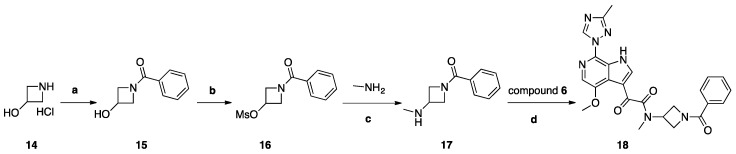
Reagents and conditions: (a) benzoyl chloride, K_2_CO_3_, H_2_O, THF, 0 °C, 2 h, r.t.; (b) MsCl, Et3N, THF, 0 °C, 1 h, r.t.; (c) H_2_O, (CH_3_)_2_CHOH, 130 °C, 48 h; (d) HATU, DIEA, DMF, 20 °C, 18 h.

**Scheme 5:**
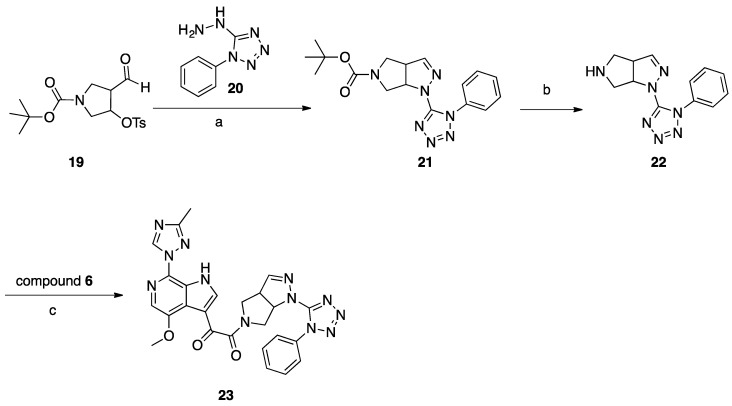
Reagents and conditions: (a) MeOH, reflux, 2 h; (b) 2 M HCl in dioxane, r.t.; (c) HATU, DIEA, DMF, r.t., 18 h.

Due to the lack of structural information on the bioactive conformation of our inhibitors and the BMS piperazine-based inhibitors at the time of design, we first used FieldTemplater (Forge, Cresset) to determine the most likely 3D conformation adopted by BMS-377806 [6], BMS-488043 [7], BMS-626529 [8], and **SC11**/**SC26** (our most potent inhibitors) [4] when complexed with the HIV-1 Env complex. The resultant bioactive conformation hypothesis (Figure 1) was then inputted into Spark (Cresset, UK) to identify nonclassical bioisosteres of the selected region. Given our previous success in using field-point methods to discover novel analogs of the central core region of our compounds, we again focused in on this region; that is, the piperazine groups of BMS-377806, BMS-488043, and BMS-626529, and the dipyrrolodine group of compounds **SC11**/**SC26**. From this analysis, three new core chemotypes were chosen for investigation, due to their calculated likelihood of being able to span a wide range of potencies. First, a common head group to be used in all of the molecules was synthesized: compound **6**, according to Materials and Methods, Scheme 1. This was subsequently used in the synthesis of compounds **SC16**, **SC18**, and **SC39** as outlined in Materials and Methods, Schemes 3–5. The high-affinity compound **SC11** was also synthesized, as previously described by Tuyishime et al. [4], and summarized in Materials and Methods, Scheme 2.

### 2.2. Potency Determination of Compounds ***SC11***, ***SC16***, ***SC18***, and ***SC39*** against Subtype B Env Pseudoviruses

Following the synthesis of compounds SC11, SC16, SC18, and SC39, we then tested them for specificity and potency against HIV-1 using the single-round infection assay [4,5]. Single-round infective HIV-1 were pseudotyped with envelopes from HIV-1 JR-CSF, HIV-1 B41 [9], or amphotropic murine leukemia virus (AMLV). HIV-1 pseudoviruses containing the aforementioned envelopes were produced, normalized to p24 content, and used to infect U87.CD4.CCR5 target cells. Infectivity was quantified by measuring luciferase activity in cell lysates (Luciferase Assay System, Promega, Madison, WI, USA) using a microplate luminometer (GloMax, Promega). The specificity of the compounds to HIV-1 Env was assessed by monitoring their effect upon the infection of AMLV Env pseudotyped recombinant HIV-1. Lastly, cytotoxicity of the compounds was determined, as outlined in Materials and Methods, up to a highest test concentration of 50 μM. The results of this analysis are shown in Table 1.

As can be seen from the Table 1, the individual compounds all had antiviral effects on HIV-1 Env pseudotyped virus in the single-round infection assay. In general, the SC compounds appear to be more effective against those viruses pseudotyped with the HIV-1_JR-CSF_. However, the median IC_50_ of the HIV-1_B41_ and HIV-1_JR-CSF_ Env pseudotyped virions are effectively spread through a range of potencies from low nanomolar to low micromolar. This potency range is perfect for investigating whether there is a correlation between affinity/kinetics and potency. 

### 2.3. Affinity and Kinetic Characterization of ***SC11***, ***SC16***, ***SC18***, and ***SC39*** for Soluble Recombinant Env Trimer

**Figure 2:**
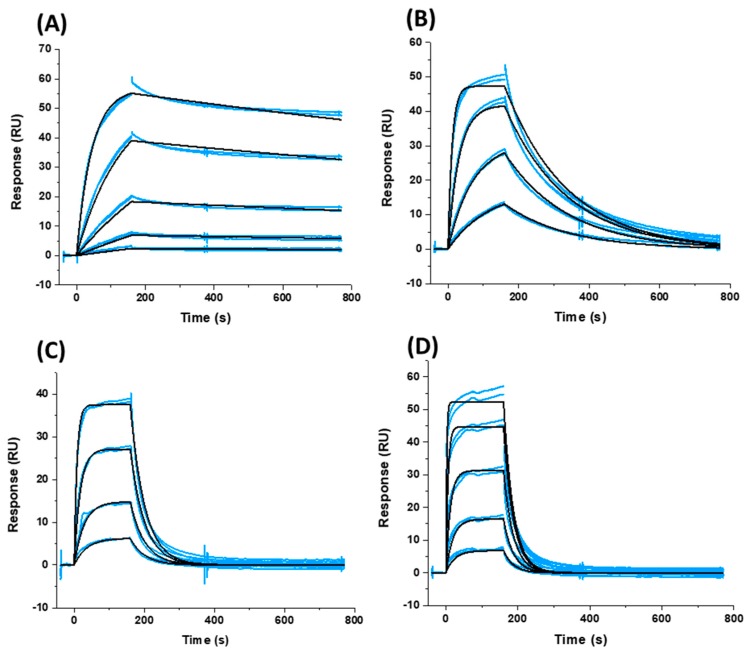
Sensorgrams depicting the interaction of (**A**) **SC11**, (**B**) **SC16**, (**C**) **SC18**, and (**D**) **SC39** with sensor chip-immobilized B41 SOSIP.664 gp140 trimer. Blue lines represent actual data, whereas black lines indicate fitting to a 1:1 interaction model. Each panel shows two injections at each concentration to demonstrate reproducibility.

Having effectively established a set of compounds with a perfect range of potencies for further correlation analysis, we next sought to characterize their interactions (affinity and kinetics) with a soluble recombinant Env trimer. To ensure consistency, we chose to utilize the B41 SOSIP.664 gp140 trimer (generously gifted to us by Dr. Andrew Ward, Scripps Institute, La Jolla, CA, USA) and to use surface plasmon resonance (SPR), so as not to have to label the reagents and to minimize consumption. Detailed conditions are given in Materials and Methods. Briefly, the B41 SOSIP.664 gp140 trimer was attached to a Sensor Chip CM5 (GE Healthcare Life Sciences, Bjorkgatan 30, Uppsala, 751 84, Sweden) via amine coupling chemistry to a final level of approximately 16,000 response units (RU) on each of eight channels of the Biacore^TM^ 8K instrument (GE Healthcare). Reference flow cells were prepared similarly using amine coupling of IgG b12 as a negative control. Compounds were injected for 160 s at 75 μL min^−1^ and with 600 s of dissociation, using the multicycle kinetics injection mode, and the surface was regenerated using a 60-s injection of 10 mM glycine pH 1.5 to ensure complete removal of bound compounds. Association and dissociation rate constants and calculated K_D_ values for binding were determined using a simple 1:1 binding model in the Biacore^TM^ 8K Evaluation software (GE Healthcare Life Sciences, Bjorkgatan 30, Uppsala, 751 84, Sweden) and from quadruplicate runs. Figure 2 shows overlaid sensorgrams from two such runs for each compound and their corresponding fits. Table 2 shows the derived parameters from the fitting of all four data sets.

**Figure 3:**
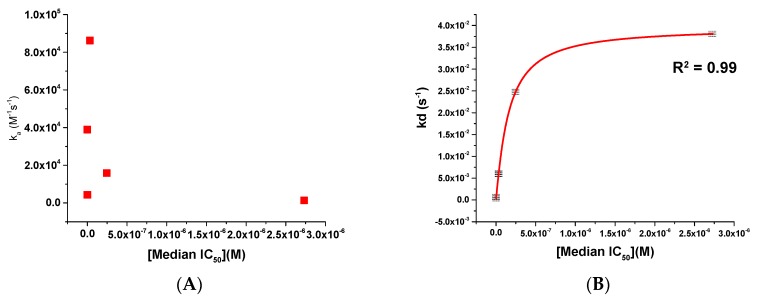
Scatter plots showing (**A**) k_a_ versus median IC_50_ and (**B**) k_d_ versus median IC_50_ for compounds BMS-626529, SC11, SC16, SC18, and SC39. The red line in panel (**B**) shows a logarithmic trend line fit to the data.

As can be seen from both Figure 2 and Table 2, there is a most striking difference between the compounds’ interactions within their kinetic profile, in particular for their off-rates. As such, we next chose to plot these kinetic parameters against the median IC_50_ to see whether or not we could establish a relationship that could be exploited. This analysis for the kinetic parameters k_a_ and k_d_ is shown in Figure 3.

### 2.4. Molecular Modeling and Computational Analysis of Compounds of ***SC11***, ***SC16***, ***SC18***, and ***SC39***

Since the original design of the SC series of compounds, including those included in this study, the structures of two BMS piperazine-based entry inhibitors complexed with the BG505 SOSIP.664 soluble trimer have been solved by Kwong et al. [10]. Therefore, to gain further insight into the potency differentials between **SC11**, **SC16**, **SC18**, and **SC39**, and to inform future design, we performed molecular modeling of these compounds into the BMS-626529-SOSIP complex structure (PDB: 5U7O) as outlined in Materials and Methods.

**Figure 4:**
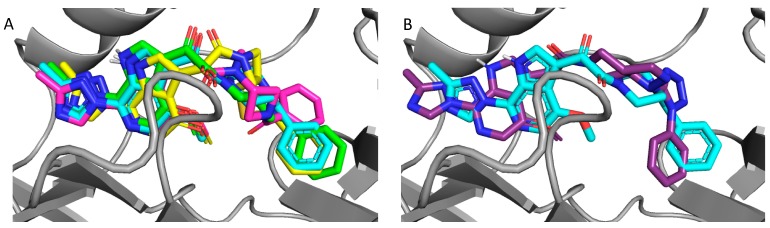
Overlay of docked compounds on the cocrystal of BMS-626529; (**A**) BMS-626529 (cyan), compound **8** (**SC11**, green), compound **13** (**SC16**, yellow), and compound 18 (**SC18**, magenta). (**B**) BMS-626529 (cyan) and compound **23** (**SC39**, purple).

Consistent with their antiviral potency, compounds **SC11**, **SC16**, and **SC18** align well with the cocrystallized BMS-626529. The triazole–azaindole moieties of these compounds adopt a very similar orientation to that of BMS-626529, whereas the terminal benzoyl moieties occupy slightly different positions (in the case of **SC18**). The octahydropyrrolo[3,4-*c*]pyrrole moiety of **SC11** effectively interacts with the α1-helix residue Trp112 and the β_20/21_ loop residue Trp427 indole rings via Pi–alkyl interactions, which may be a reason for the observed potency of **SC11** (median IC_50_ of ~2 nM, predicted binding energy of −13.23 kcal/mol). For compounds **SC16** and **SC18**, the smaller azetidine may provide fewer interactions with those residues and hence the weaker activities compared to **SC11** (**SC16** median IC_50_ = 33 nM, with a predicted binding energy of −12.26 kcal/mol; **SC18** median IC_50_ = 246 nM, with a predicted binding energy of −12.17 kcal/mol). For the weaker compound **SC39**, the kink introduced by the presence of the terminal phenyl–tetrazole pushes the triazole–azaindole out of the binding site (compared to BMS-626529), resulting in a slightly different binding mode (Figure 4B), which may be the reason for the reduced activity (median IC_50_ = 2.73 μM, with a predicted binding energy of −11.36 kcal/mol).

**Figure 5:**
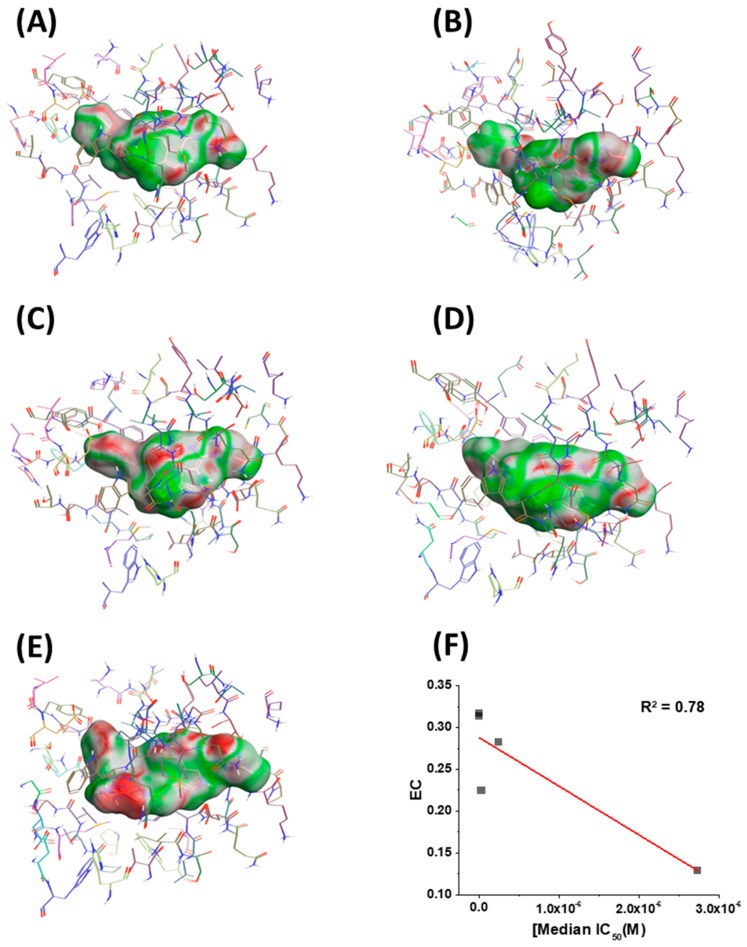
Electrostatic complementarity (EC) of the binding site in BG505 SOSIP.664 gp140 trimers and (**A**) BMS-626529, (**B**) SC11, (**C**) SC16, (**D**) SC18, and (**E**) SC39. (**F**) EC score plotted versus median IC_50_. Red line shows a linear trend line. Regions of the ligand surface where there is perfect electrostatic complementarity with the protein are colored green, while the regions where there is a perfect electrostatic clash are colored red.

Having in hand models of compounds bound to their target, we next sought to identify any other correlations that could inform future design. Therefore, we chose to look at the electrostatic complementarity between the compounds and the binding site on the BG505 SOSIP.664 protein using Flare v2.0 (Cresset, Litlington, Cambridgeshire, UK; http://www.cresset-group.com/flare/). Electrostatic complementarity (EC) maps are based on a calculation of electrostatic potentials for the ligand and the protein on the surface of the ligand. These potentials are then added together, normalized, and scaled. Regions of the ligand surface where there is perfect electrostatic complementarity with the protein are colored green, while the regions where there is a perfect electrostatic clash are colored red. Flare v2.0 can also compute several electrostatic complementarity summary scores for quantification and comparison of the EC. Therefore, we performed this analysis on our derived models for the **SC11**, **SC16**, **SC18**, and **SC39** complexes (Figure 5) as well as for the BMS-626529 complex. We also chose to plot the first computed score for correlation analysis with potency. This score is effectively the average value of that score over the surface of the ligand (Figure 5F). As can be seen from this analysis, each ligand has different electrostatic complementarity with the binding site on Env. Moreover, when quantified and plotted against median IC_50_, there exists a reasonable linear correlation between these values; that is, the higher the EC, the higher the potency. Now identified, this correlation can be integrated seamlessly into our computational design workflow for future compounds in this inhibitor class.

## 3. Material and Methods

### 3.1. Compounds

BMS-626529 was purchased from MedChemExpress (Monmouth Junction, NJ, USA). SC compounds were synthesized de novo as outlined below. All available QC data for the SC compounds can be found in the supplemental data file.

#### Synthesis of Common Intermediate **6** (Scheme 1)

A solution of compound **1** (30 g, 0.13 mol) in THF (1.5 L) was cooled to −78 °C. Vinylmagnesium in THF (508 mL, 1N, 0.51 mol) was added dropwise to the above solution and the temperature was maintained below −40 °C. The mixture was stirred for 3 h at −78 °C before being quenched with saturated aqueous NH_4_Cl solution. The layers were separated, the aqueous layer was extracted with EtOAc (3 × 500 mL), and the combined organic layer was washed with brine and concentrated. The crude product was purified by silica gel column chromatography to obtain compound **2** (5 g, 17%). Compound **2** (4.0 g, 17.2 mmol) was dissolved in freshly prepared NaOMe (20 mL). CuI (3.2 g, 17.2 mmol) was added and the mixture was microwave-irradiated at 110 °C and stirred for 3 h. After reaction completion, EtOAc and H_2_O were added, then the suspension was filtered and the filtrate was separated. The organic layer was concentrated to give crude product, which was purified by column chromatography to give compound **3** (1.8 g, 45%). A mixture of compound **3** (0.7 g, 3.8 mmol), 3-methyl-1,2,4-triazole (6.4 g, 76.8 mmol), copper powder (0.49 g, 7.68 mmol), and KOH (0.43 g, 7.68 mmol) was heated to melt at 170–175 °C while being protected by N_2_ atmosphere_._ After consumption of the starting material, EtOAc and H_2_O were added to the mixture, and the suspension was filtered and the filtrate was collected. The organic layer was concentrated to give the crude product, which was purified by column chromatography to give compound **4** (0.23 g, 26%). A solution of compound **4** (0.23 g, 1 mmol) in THF (12 mL) was cooled to −10 °C, and EtMgBr (0.47 g, 3.5 mmol) was added dropwise at −10 °C, followed by the addition of pyridine (0.3 mL). The slurry was cooled to −45 °C, and the ethyl 2-chloro-2-oxoacetate (0.55 g, 4 mmol) was added dropwise at −45 °C. The temperature was allowed to rise to −10 °C and the mixture was stirred for 1h. After reaction completion, the mixture was quenched with IPA (2 mL) and H_2_O (20 mL). The mixture was extracted with EtOAc, and the organic layer was washed with brine and concentrated to the give crude product which was purified by column chromatography to give compound **5** (0.17 g, 52%). Compound **5** (0.17 g, 0.52 mmol) was dissolved in MeOH (5 mL). NaOH (0.041 g, 1.03 mmol) and H_2_O (2 mL) were added and the mixture was stirred at 25 °C for 6 h. HCl (1N) was added to adjust the pH to 6, and the mixture was concentrated to give compound **6** (0.29 g) as crude product.

#### Synthesis of Compound **8** (**SC11**, Scheme 2)

Compound **8** (**SC11**) was synthesized following the procedures described by Tuyishime et al. [4].

#### Synthesis of Compound **13** (**SC16**, Scheme 3)

Compound **9** (2 g, 10 mmol) was dissolved in DCM (30 mL). NMM (2 g, 20 mmol) was added, the mixture was cooled to 0 °C, and MsCl (1.37 g, 12 mmol) was added dropwise. After addition, the mixture was stirred at 25 °C for 16 h. After reaction completion, H_2_O was added, and the mixture was extracted with DCM, the combined DCM layers were combined and evaporated, and the residue was purified by silica gel column chromatography to obtain compound **10** (1.8 g, 68%). Compound **10** (1.8 g, 6.79 mmol) was dissolved in DMF (10 mL), K_2_CO_3_ (1.87 g, 14 mmol), and benzothioic S-acid (1.12 g, 8.15 mmol) were added. The mixture was heated to 80 °C for 2 h. After reaction completion, H_2_O was added, and the mixture was extracted with EtOAc. The combined EtOAc layers were combined and washed with H_2_O and brine, then concentrated under vacuum to give crude product which was purified by silica gel column chromatography to obtain compound **11** (2 g, 96%). TFA (3 mL) in DCM (20 mL) was added to compound **11** (2 g, 6.5 mmol) and the mixture was stirred at 25 °C for 3 h, and the mixture was concentrated to give compound **12** (2.3 g, crude) as a TFA salt. To a solution of compound **12** (0.83 g, 0.4 mmol) in DMF (3 mL), DIEA (0.085 g, 0.66 mmol) was added, followed by the intermediate compound **6** (0.1 g, 0.33 mmol) and HATU (0.15 g, 0.4 mmol). The mixture was stirred at 25 °C for 2 h. The reaction mixture was then concentrated under reduced pressure to remove DMF. The residue was partitioned between with EtOAc (10 mL) and saturated NaHCO_3_ (10 mL). The combined organic layers were dried over Na_2_SO_4_, filtered, and concentrated. The crude product was purified by preparative HPLC to give compound **13** (**SC16**) (0.032 g, 20%).

#### Synthesis of Compound **18** (**SC18**, Scheme 4)

Compound **14**, 2 g (0.018 mol), was dissolved in 30 mL of THF/H_2_O (2:1) and cooled to 0 °C. To this solution, 6.06 g (0.043 mol) of K_2_CO_3_ was added, and a solution of (2.823 g, 0.0198 mol) of benzoyl chloride in 5 mL of dry THF was added dropwise. The reaction mixture was stirred at room temperature for 2 h and poured into 100 mL of a 5% solution of NaHCO_3_. The mixture was extracted with DCM (2 × 30 mL), and the organic layer was dried with Na_2_SO_4_ and concentrated to yield 1.2 g (40%) of compound **15** as colorless crystals. Compound **15**, 1 g (0.0054 mol), was dissolved in 10 mL of THF and 0.877 (0.0085 mol) of TEA was added. The mixture was cooled to 0° C, and 0.909 g (0.0077 mol) of mesyl chloride was added dropwise. The reaction mixture was stirred for 1 h at room temperature and poured into 100 mL of water. The mixture was extracted with DCM (3 × 30 mL), and the combined organic layers were washed with 20 mL of brine, dried over Na_2_SO_4_, and concentrated to yield 1.2 g (80%) of compound **16** as white crystals. Compound **16** (1.2 g, 0.0048 mol) was dissolved in 10 mL of IPA, then 7.5 g of 40% aqueous methylamine solution was added and the obtained mixture was stirred and heated in a sealed tube for 48 h at 130° C. The reaction mixture was poured into 100 mL water and extracted with DCM (5 × 20 mL). Combined extracts were dried over Na_2_SO_4_ and concentrated. The crude material was purified by column chromatography using a mixture of DCM/MeOH (98:2), then 97:3 as the eluent and fractions with Rf = 0.15 (DCM/MeOH 95:5) were collected to yield 370 mg (40%) of **17** as a colorless oil. A solution of acid **6** (69 mg, 0.23 mmol), HATU (261 mg, 0.68 mmol), and DIPEA (118 mg, 0.91 mmol) in dry DMF (10 mL) was stirred for 1 h. Then, amine **17** (87 mg, 0.45 mmol) was added. The reaction mixture was stirred at 20 °C for 18 h. Then, water (100 mL) with saturated NaHCO_3_ (10 mL) was added. The mixture was with EtOAc (3 × 30 mL). Combined extracts were dried over Na_2_SO_4_ and concentrated. The crude material was purified by column chromatography using the mixture DCM/MeOH (98:2) as the eluent to yield 27 mg (25%) of compound **18** (**SC18**) as a pale-yellow solid.

#### Synthesis of Compound **23** (**SC39**, Scheme 5)

A mixture of aldehyde **19** (1.1 g 2.98 mmol) and hydrazine **20** (0.42 g, 2.38 mmol) in 20 mL of methanol was refluxed for 2 h, concentrated, and purified with column chromatography (DCM/MeOH 99:1) to give 214 mg (26%) of compound **21** as a white solid. To 5 mL of a 2 M solution of HCl in dry dioxane, compound **21** (140 mg, 0.39 mmol) was added at room temperature. The reaction mixture was stirred overnight at room temperature, then 20 mL of dry ether was added, and the precipitated solid was filtered off and washed with dry ether (2 × 10 mL). The amine **22** HCl salt (64 mg, 57%) was obtained as a white solid. A mixture of acid **6** (101 mg, 0.33 mmol), amine hydrochloride **22** (98 mg, 0.33 mmol), DIPEA (217 mg, 1.66 mmol), and HATU (382 mg, 0.99 mmol) in 5 mL of dry DMF was stirred for 18 h at room temperature. The reaction mixture was poured into 100 mL of water and extracted with DCM (3 × 30 mL), and the combined organic layers were washed with brine, dried with Na_2_SO_4_, and concentrated. The residue was suspended in water and was filtered off and recrystallized from 3 mL of EtOH to give compound **23** (**SC39**; 16 mg, 9%) as a pale-yellow solid. 

### 3.2. Cells

Human embryonic kidney 293T cells (a gift from Dr. Irwin Chaiken, Drexel University, Philadelphia, PA, USA) were cultured in Dulbecco’s Modified Eagle’s Medium (DMEM), 10% FBS, 100 U/mL penicillin, 100 μg/mL streptomycin, and 2 mM l-glutamine. Human astroglioma U87 cells stably expressing CD4/CCR5 or CD4/CXCR4 (obtained from Prof. Hongkui Deng, Peking University, and Prof. Dan Littman, New York University, USA, through the AIDS Research and Reference Reagent Program, Division of AIDS, NIAID, NIH [11,12]) were cultured in DMEM supplemented with 10% FBS, 100 U/mL penicillin, and 100 μg/mL streptomycin and 2 mM l-glutamine, 300 μg/mL G418 (Thermo Scientific, Waltham, MA, USA), and 1 μg/mL puromycin (Thermo Scientific). Cells were incubated continuously, unless otherwise stated, at 37 °C in a humidified 5% CO_2_/95% air environment. 

### 3.3. Proteins

The soluble cleaved trimer B41 SOSIP.664 gp140 was a generous gift from Dr. Andrew Ward (Scripps Institute, La Jolla, CA, USA); IgG b12 anti-HIV-1 gp120 was obtained through the NIH AIDS Reagent Program, Division of AIDS, NIAID, NIH; anti-HIV-1 gp120 monoclonal antibody (IgG1 b12) was obtained from Dr. Dennis Burton and Carlos Barbas. The overproduction and purification of p24 (HIV-1 capsid [CA]) were performed as previously described [13]. Briefly, a vector containing C-terminally His-tagged HIV-1_NL4-3_ CA (a gift from Dr. Eric Barklis, Oregon Health and Science University, Portland, OR, USA) was transformed into BL21-Codon Plus (DE3)-RIL Competent Cells (Agilent Technologies, Wilmington, DE, USA) and grown up in autoinduction ZYP-5052 medium overnight with shaking (225 rpm) at 30 °C [14]. Bacterial cultures were spun down at 7000 rpm, and the supernatant was discarded. Cell pellets were resuspended in 40 mM Tris–HCl pH 8.0, 500 mM NaCl, and 3 mM imidazole and lysed via sonication. The resultant supernatant was clarified and immediately applied to a Talon cobalt resin affinity column (Clonetech Laboratories, Mountain View, CA, USA). Bound CA was washed and eluted using increasing concentrations of imidazole. Purified CA-H6 was dialyzed overnight into 20 mM Tris–HCl pH 8.0 at 4 °C, concentrated to 120 μM, flash frozen in liquid nitrogen, aliquoted, and stored at −80 °C. 

### 3.4. Production of Pseudotyped Viruses

Single-round infectious envelope-pseudotyped luciferase-reporter viruses were produced by dual transfection of two vectors (3 μg of vector 1 and 4 μg of vector 2) in 6-well plated 293T cells (1 × 10^6^ cells/well) [12]. Vector 1 is an envelope-deficient HIV-1 pNL4-3-Luc+R-E plasmid which carries the luciferase reporter gene [15]. Vector 2 is a plasmid expressing either the HIV-1 gp160 Env, vesicular stomatitis virus glycoprotein (VSV-G), or amphotropic murine leukemia virus Env (AMLV) [16,17]. Transfections of these vectors were carried out via calcium phosphate (ProFection Mammalian Transfection System, Promega, Madison, WI, USA) for 5 h. Following the 5 h transfection incubation, DNA-containing medium was removed and cells were washed with DMEM and replenished with fresh culture media. Supernatants containing pseudovirus were collected 72 h post-transfection, clarified, filtered, aliquoted, and stored at −80 °C. 

### 3.5. ELISA-Based Quantification of p24 Content

ELISA plate was coated with 50 ng of mouse anti-p24 (Abcam, ab9071, Cambridge, MA, USA) for overnight at 4 °C, blocked with 3% BSA for 2 h at room temperature, and washed with 0.5% Tween in PBS. Pseudoviral stocks were lysed with 0.1% Triton X-100 (Sigma-Aldrich, St. Louis, MO, USA) at 37 °C for 1 h and added to the plate overnight at 4 °C. Simultaneously, p24 protein (produced, purified, and validated as described above) was added for the generation of a standard curve. Following the overnight incubation, the plate was washed with PBST and 1:5000 dilution of rabbit anti-p24 (Abcam, ab63913) was added for 2 h at room temperature. After washing the unbound rabbit anti-p24 off the plate with PBST, goat anti-rabbit-HRP at a 1:5000 dilution was added for 1 h at room temperature. The plate was then extensively washed with PBST. Subsequently, a solution of 0.4 mg/mL *O*-phenylenediamine in a phosphate–citrate buffer with sodium perborate (Sigma-Aldrich) was added and incubated in the dark for 30 min. Optical densities were then obtained at 450 nm in a Multiskan™ GO Microplate Spectrophotometer (Thermo Scientific). 

### 3.6. Single-Round Infection Assay

The details of the single-round HIV-1 infection assay have been published previously [15,18,19]. Briefly, U87.CD4.CCR5/CXCR4 (1.2 × 10^4^ cells/well) target cells were seeded in 96-well luminometer-compatible tissue culture plates (Greiner Bio-One, Monroe, NC, USA). After 24 h, compound, DMSO (vehicle control for compounds, Sigma) was mixed with pseudotyped viruses (normalized to p24 content), and the mixture was added to the target cells and incubated for 48 h at 37 °C. Following this, the media was removed from each well, and the cells were lysed by the addition of 50 μL of luciferase lysis buffer (Promega) and one freeze–thaw cycle. A GloMax 96 microplate luminometer (Promega) was used to measure the luciferase activity of each well after the addition of 50 μL of luciferase assay substrate (Promega). 

### 3.7. Cellular Toxicity

The viability of the U87.CD4.CCR5/CXCR4 cells was determined by the Cell Counting Kit-8 cell proliferation and cytotoxicity assay (Dojindo Molecular Technologies, Rockville, MD, USA) as per the manufacturer’s instructions. U87.CD4.CCR5/CXCR4 cells were seeded at a density of 1.2 × 10^4^ cells/well in 96-well tissue culture plates (Olympus plastics) 24 h before treatment. Cells were treated with compound (1 mM–0.1 μM) or DMSO (Sigma) for 48 h at 37 °C. Subsequently, 10 μL of the CCK-8 solution was added to each well and allowed to incubate at 37 °C for 4 h before absorbance at 450 nm was measured in a Multiskan™ GO Microplate Spectrophotometer (Thermo Scientific). Untreated cells were used as a medium control and 0.1% SDS-treated cells served as a positive control. The 50% cytotoxicity (CC_50_) value was defined as the concentration of the compounds that reduced the absorbance of treated cells by 50% as compared with control cells.

### 3.8. SPR Direct Interaction Analysis

Binding kinetics and affinity of the compound inhibitors were measured using SPR-based binding assays that were performed with a Biacore^TM^ 8K instrument (GE Healthcare). The B41 SOSIP.664 gp140 trimer was attached to a Sensor Chip CM5 (GE Healthcare) via amine coupling chemistry. SOSIP Env trimer was diluted into 10 mM sodium acetate pH 5.0 to a concentration of 50 μg mL^−1^ and injected for 10 min over the active flow cell of an EDC/NHS (400 mM *N*-ethyl-*N*-(3-dimethylaminopropyl)-carbodiimide hydrochloride and 100 mM *N*-hydroxysuccinimide) reactive surface to a final level of approximately 16,000 RU on each of 8 channels. Non-crosslinked reactive esters were blocked with 1 M ethanolamine. Reference flow cells were prepared similarly using amine coupling of IgG b12 as a negative control. The running buffer used was PBS-P^+^ (20 mM phosphate buffer at pH 7.4, with 27 mM KCl, 137 mM NaCl, and 0.05% surfactant P20) for amine coupling, with the addition of 3% DMSO for compound binding assays. Compounds were injected for 160 s at 75 μL min^−1^, and 600 s of dissociation, using the multicycle kinetics injection mode. Data was collected at a rate of 10 Hz. The surface was regenerated using a 60-s injection of 10 mM glycine pH 1.5 to ensure complete removal of bound compounds. Biacore^TM^ 8K Evaluation software was used for kinetic analysis. Raw sensorgram data were reference-subtracted and blank-buffer-subtracted before kinetic and affinity analysis to account for nonspecific binding and injection artifacts. Association and dissociation rate constants and calculated K_D_ values for binding were determined using a simple 1:1 binding model from quadruplicate runs.

### 3.9. Molecular Modeling 

#### 3.9.1. Bioactive Conformation Hypothesis Prediction

Generation of a hypothesis for the conformation adopted by BMS-377806, BMS488043, BMS-626529, and SC11 was achieved using FieldTemplator in Forge, version 10.5 (Cresset^®^, Litlington, Cambridgeshire, UK, http://www.cresset-group.com/forge/). Briefly, each of the four compounds was conformationally populated using Cresset’s Xedex conformational search method (embedded within Forge). The 3D field point pattern for each conformation of each template molecule was then calculated and used to cross-compare to each other in field/shape space, with no dependence on chemical structure. Using 3D field point patterns, the conformations of the compounds were exhaustively compared in a pairwise fashion until field point patterns common to all four molecules were identified. The resulting templates (i.e., binding mode hypotheses) each consist of one conformation of each of the four molecules (quartets). The quartets from the FieldTemplater experiment using the compounds were visually inspected, and one conformation of BMS-626529 was selected as the query molecule for the Spark bioisosteric replacement experiments (Spark, version 10.5, Cresset^®^, Litlington, Cambridgeshire, UK; http://www.cresset-group.com/spark/). The selected conformation of BMS-626529 appeared in several of the top-scoring templates.

#### 3.9.2. Docking Simulations

BMS-626529, SC11, SC16, SC18, and SC39 were prepared and then energy-minimized using the MMFF94 Force Field (ChemBio3D Ultra 15.0) with a root mean squared (RMS) gradient of 0.01 and number of alterations of 10,000. The minimized structures were then saved as PDB files for the docking simulations. The Autodock tools graphical interface (MGtools 1.5.7rc1) [15,16] was then used to prepare the compounds for docking.

The crystal structure of BMS-626529 bound to BG505 SOSIP.664 trimer (PDB code: 5U7O) [10] was downloaded and prepared by the Autodock tools graphical interface (MGtools 1.5.7rc1), where nonpolar hydrogens were merged, Kollman charges were added, and Gasteiger charges were calculated. The grid box for the docking search was centered on the cocrystalized ligand BMS-626529 and set to 40, 56, and 40 points for the x, y, and z dimensions, respectively, with a spacing grid of 0.375 Å. The AutoGrid 4.2 algorithm [20,21] was used to evaluate the binding energies between the compounds and the protein and to generate the energy maps for the docking run. For high-accuracy mode docking of the compounds with the protein, the maximum number of evaluations (25 Å~10^6^) was used. Fifty runs were generated for each compound by using the Autodock 4.2 Lamarckian genetic algorithm [20,21] for the searches. Cluster analysis was performed on docked results, with a root mean square tolerance of 0.5 Å. The docking protocol was validated by docking the prepared BMS-626529 structure, and this generated only one stable cluster of poses. The lowest energy pose was very similar to the cocrystalized BMS-626529, with an RMS value of 0.67. The calculated binding energy (ΔG binding) and predicted Ki values for the compound−protein complexes were recorded. 

## 4. Conclusions

In this study, we have generated a series of four compounds with ranges of antiviral potency against two subtype B Env pseudotyped HIV-1 viruses in the low nanomolar to the low micromolar range. We have established a novel SPR assay to determine the kinetic parameters and correlated the off-rates with antiviral potency. Lastly, we have identified a relationship between electrostatic complementarity of the ligands with their respective binding site on Env and their potency. These relationships can now be incorporated into our computational design and experimental workflows to greatly facilitate our entry inhibitor design efforts and fulfill our overarching goal. 

## Figures and Tables

**Table 1:** Specificity and potency of compounds against HIV-1_JR-CSF_, HIV-1_B41_, and AMLV-pseudotyped HIV-1. N.A. = not active. The chemical structures were drawn with ChemDraw Ultra 15.0.

Compound	IC_50_ JR-CSF (μM)	IC_50_ B41 (μM)	IC_50_ AMLV (μM)	Median IC_50_ (μM)	CC_50_ (μM)
**BMS-626529**	0.00006 ± 0.000005	0.00005 ± 0.000006	N.A.	0.000055	>50
Compound **8** (**SC11**) 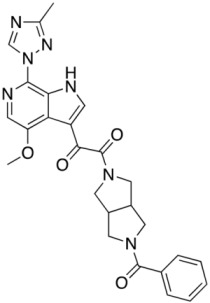	0.0006 ± 0.0001	0.002 ± 0.0002	N.A.	0.001	
Compound **13** (**SC16**) 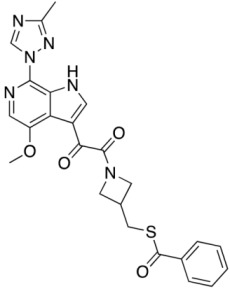	0.029 ± 0.008	0.037 ± 0.005	N.A.	0.033	
Compound **18** (**SC18**) 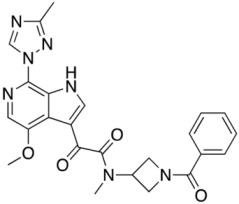	0.132 ± 0.004	0.360 ± 0.012	N.A.	0.246	
Compound **23** (**SC39**) 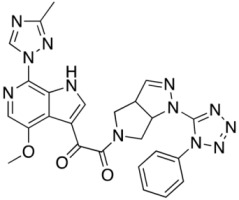	0.143 ± 0.052	5.32 ± 0.26	N.A.	2.73	

**Table 2:** Kinetics and affinity of BMS-626529 and the SC compounds binding to soluble, cleaved recombinant Env.

Compound	k_a_ (M^−1^ s^−1^)	k_d_ (s^−1^)	K_D_
**BMS-626529** *	3.89 × 10^4^	5.9 × 10^−4^	15.2 nM
**SC11**	4.33 × 10^3^	2.87 × 10^−4^	66.3 nM
**SC16**	8.62 × 10^4^	5.98 × 10^−3^	69.3 nM
**SC18**	1.59 × 10^4^	2.48 × 10^−2^	1.56 μM
**SC39**	1.39 × 10^3^	3.81 × 10^−2^	27.4 μM

* The sensorgrams for BMS-626529 binding to soluble, cleaved recombinant Env are shown in Supplementary Materials.

## Supplementary Materials

The following are available online, SPR supplemental methods and Figure S1: QC data for the SC compounds.
