# Supplementary material for: Kinetic Characterization of Novel HIV-1 Entry Inhibitors: Discovery of a Relationship between Off-Rate and Potency
Source: Molecules. 2018 Aug 3;23(8):1940. doi: 10.3390/molecules23081940 (PMC6222832; doi:10.3390/molecules23081940)
Supplement: Supplementary file 1 [file molecules-23-01940-s001.pdf]

SPR interaction analysis: BMS-626529

Binding assays using BMS-626529 were performed on a ProteOn XPR36 SPR Protein Interaction Array System (Bio-Rad Laboratories, Hercules, CA). The instrument temperature was set at 25°C for all kinetic analyses. ProteOn GLH sensor chips were preconditioned with two short pulses each (10 seconds) of 50 mM NaOH, 100 mM HCl, and 0.5% sodium dodecyl sulfide. Then the system was equilibrated with PBS-T buffer (20 mM sodium phosphate, 150 mM NaCl, and 0.005% polysorbate 20, pH 7.4). The surface of a GLH sensorchip was activated with a 1:100 dilution of a 1:1 mixture of 1-ethyl-3-(3-dimethylaminopropyl) carbodiimide hydrochloride (0.2 M) and sulfo-*N*-hydroxysuccinimide (0.05 M). Immediately after chip activation, the HIV-1 B41 SOSIP.664 gp140 trimer, purified as outlined in Pugach et al.,[1] was prepared at a concentration of 100 μg/ml in 10 mM sodium acetate, pH 5.0 and injected across ligand flow channels for 15 min at a flow rate of 30 μl/min. Then, after unreacted protein had been washed out, excess active ester groups on the sensor surface were capped by a 5 minutes injection of 1 M ethanolamine HCl (pH 8.0) at a flow rate of 5 μl/min. This resulted in a ligand density of 13,600 RU (THeoretical R_{max} = ~40 RU). A reference surface was similarly created by immobilizing a non-specific protein (IgG b12 anti HIV-1 gp120; was obtained through the NIH AIDS Reagent Program, Division of AIDS, NIAID, NIH: Anti-HIV-1 gp120 Monoclonal (IgG1 b12) from Dr. Dennis Burton and Carlos Barbas) and was used as a background to correct non-specific binding.

To prepare BMS-626529 for direct binding analysis, compound stock solutions, along with 100% DMS, and totaling 30μl was made to a final volume of 1 ml by addition of sample preparation buffer (PBS, pH 7.4). Preparation of analyte in this manner ensured that the concentration of DMSO was matched with that of running buffer with 3% DMSO. Serial dilutions were then prepared in the running buffer (PBS, 3% DMSO, 0.005% polysorbate 20, pH 7.4) and injected at a flow rate of 100 μl/min, for a 1 minute association phase, followed by up to a 10 minutes dissociation phase using the "one shot kinetics" capability of the Proteon instrument.[2] Data were analyzed using the ProteOn Manager Software version 3.0 (Bio-Rad). The responses of a buffer injection and responses from the reference flow cell were subtracted to account for the nonspecific binding and injection artifacts. The kinetic rate parameters, derived from a minimum of four experiments, were calculated in ProteOn Manager Version 3.1.0.6 (Bio-Rad, Hercules, CA), by fitting to a simple Langmuir 1:1 binding model**.** The average of the on- and off-rates were used to determine the equilibrium dissociation constant, K_D.

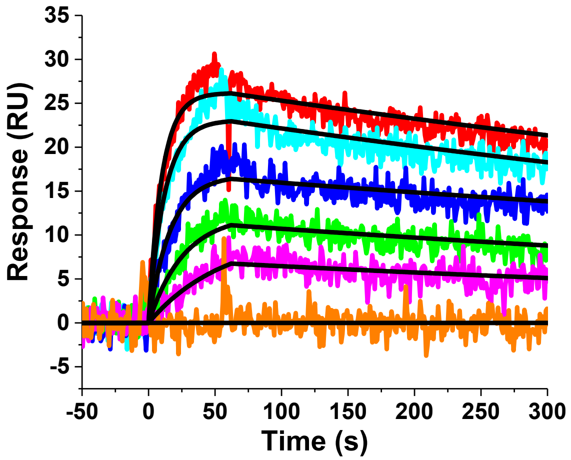

Supplemental Figure 1. Representative sensorgrams depicting the BMS-626529 interaction with soluble, cleaved Env trimer. Coloured lines depict actual data, whereas black lines show fitting to a simple 1:1 binding model. BMS-626529 was injected over the Env surfaces at 5, 2.5, 1.25, 0.625, 0.3125, and 0 μM concentrations.

1. Pugach, P.; Ozorowski, G.; Cupo, A.; Ringe, R.; Yasmeen, A.; de Val, N.; Derking, R.; Kim, H. J.; Korzun, J.; Golabek, M.; de Los Reyes, K.; Ketas, T. J.; Julien, J. P.; Burton, D. R.; Wilson, I. A.; Sanders, R. W.; Klasse, P. J.; Ward, A. B.; Moore, J. P., A Native-Like SOSIP.664 Trimer Based on an HIV-1 Subtype B env Gene. *J Virol* **2015,** *89* (6), 3380-95.
2. Bravman, T., et al., *Exploring "one-shot" kinetics and small molecule analysis using the ProteOn XPR36 array biosensor.* Anal Biochem, 2006. **358**(2): p. 281-8.

QC data for compounds

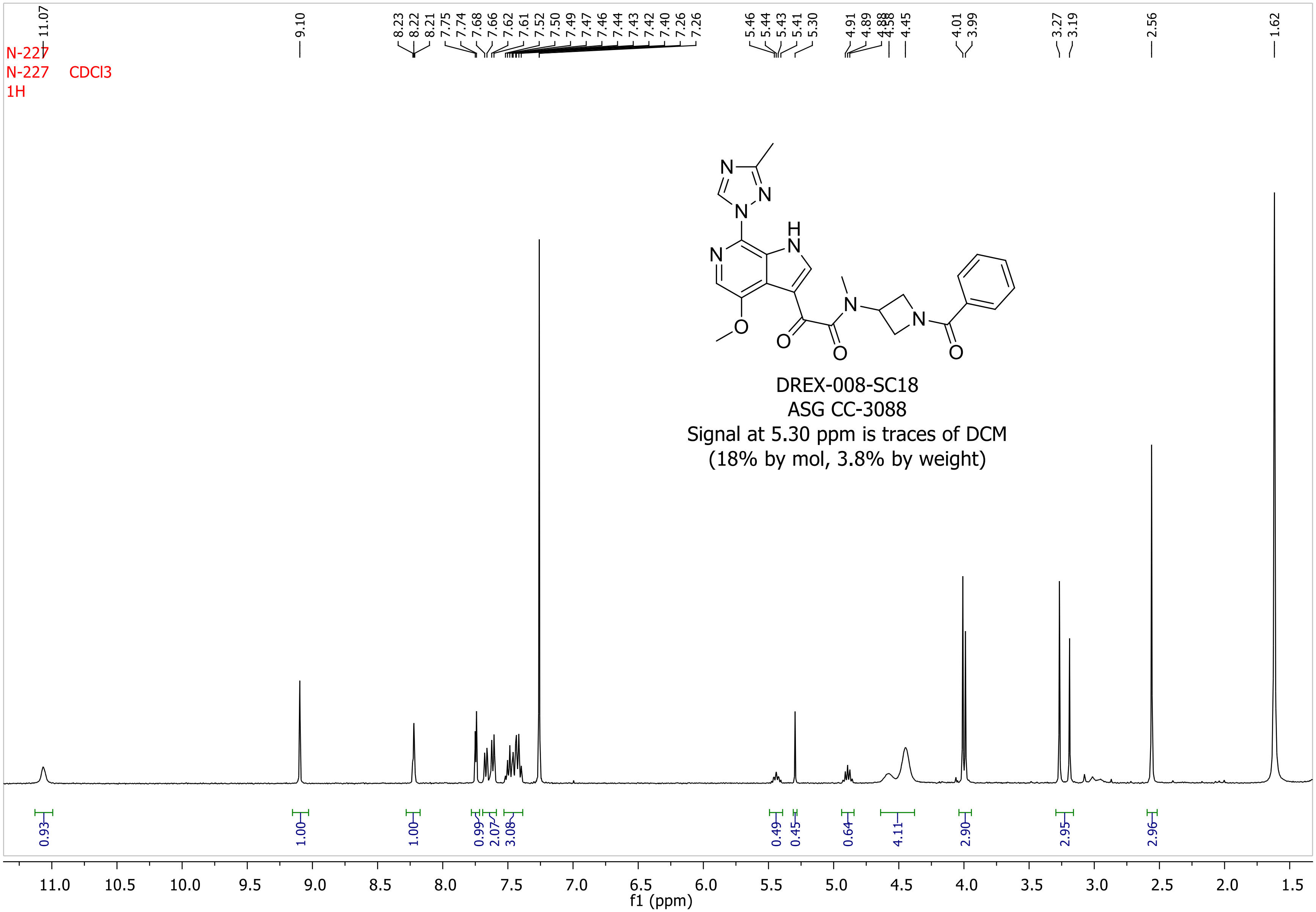

LCMS. SC18

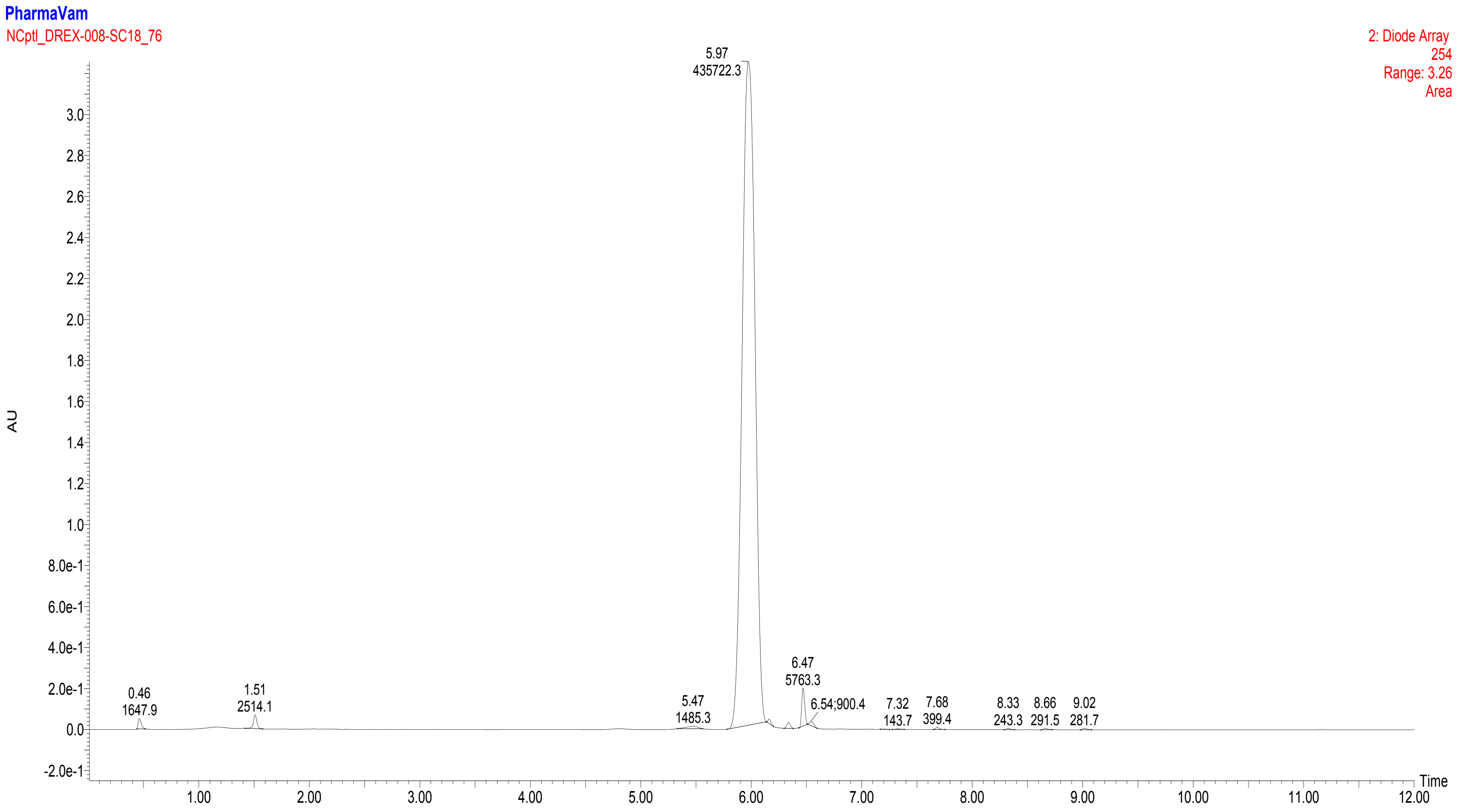

PharmaVam
NCptl_DREX-008-SC18_76
2: Diode Array
254
Range: 3.26
Area
AU
Time
5.97
435722.3
0.46
1647.9
1.51
2514.1
5.47
1485.3
6.47
5763.3
6.54;900.4
7.32
143.7
7.68
399.4
8.33
243.3
8.66
291.5
9.02
281.7

LCMS. SC18.

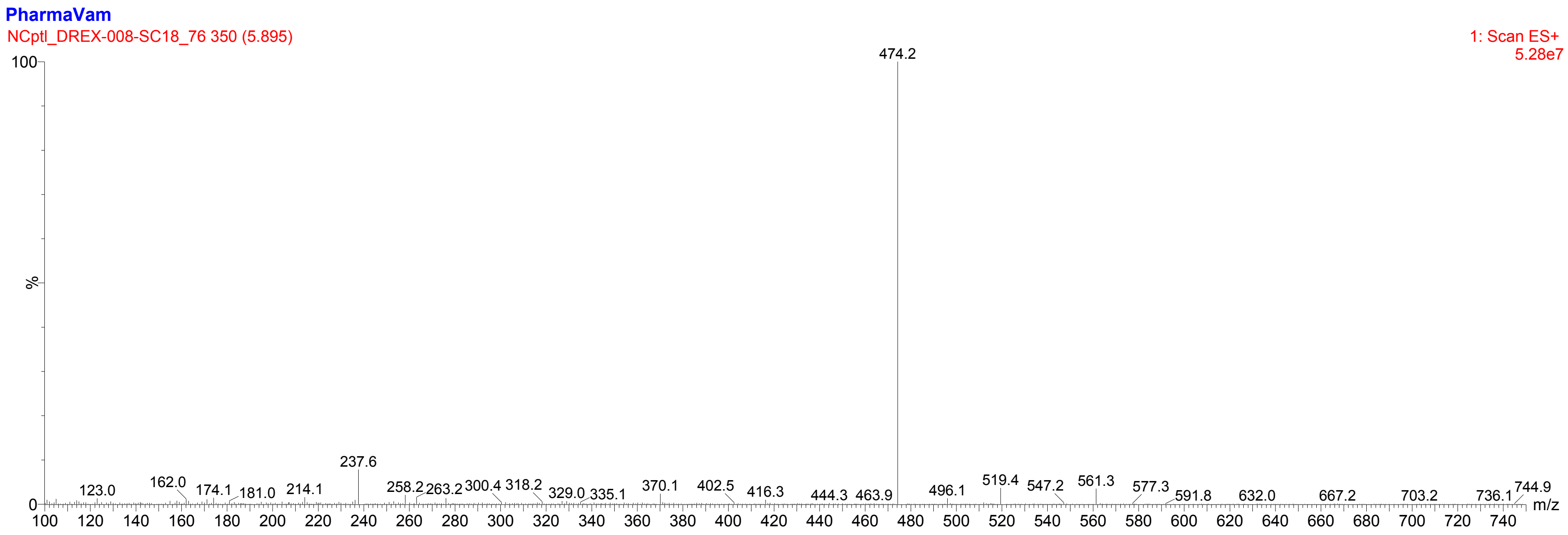
PharmaVam
NCptl_DREX-008-SC18_76 350 (5.895)
1: Scan ES+
5.28e7
474.2
%
237.6
123.0
162.0
174.1
181.0
214.1
258.2
263.2
300.4
318.2
329.0
335.1
370.1
402.5
416.3
444.3
463.9
496.1
519.4
547.2
561.3
577.3
591.8
632.0
667.2
703.2
736.1
744.9
m/z

SC39

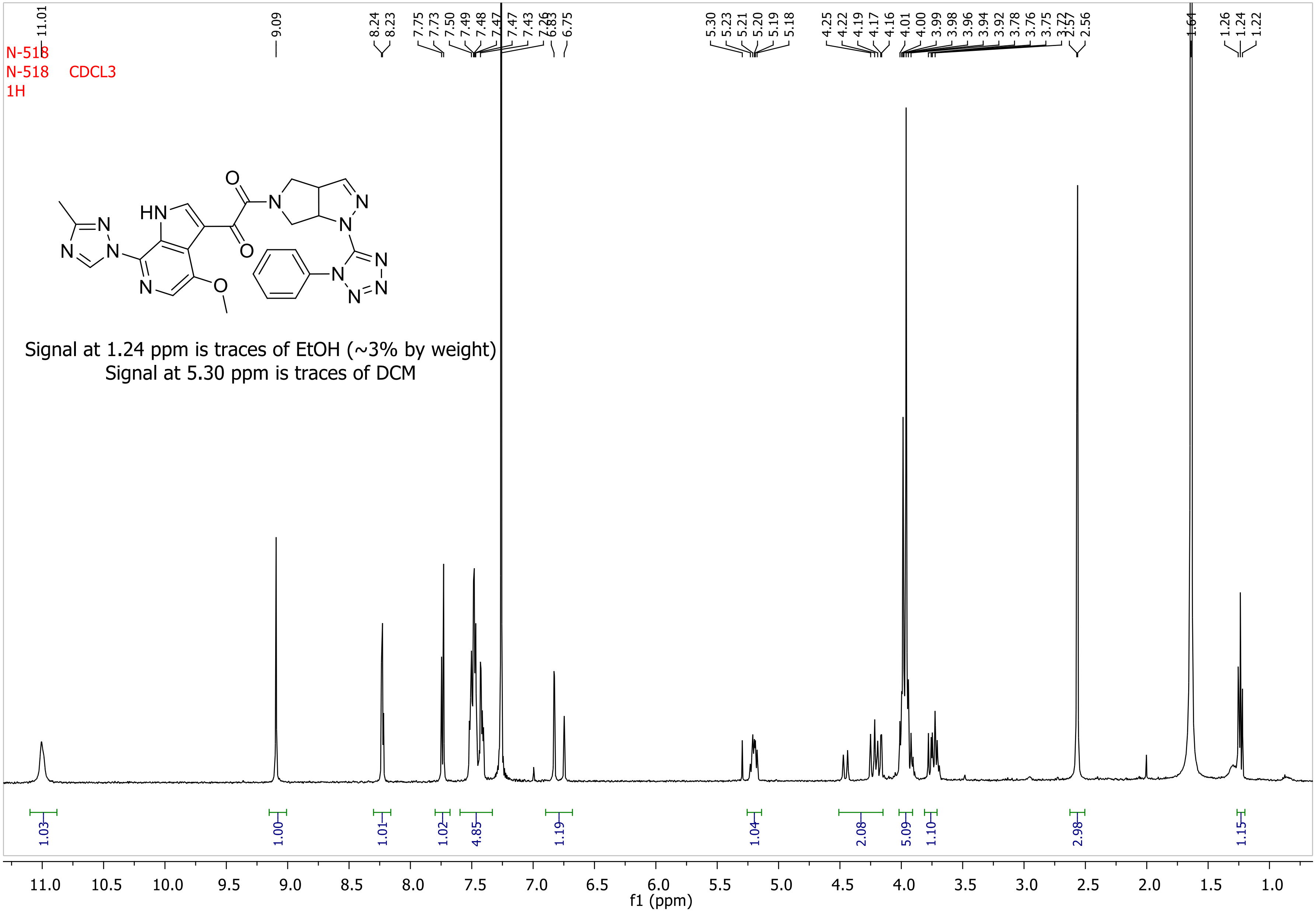

N-518
N-518 CDCL3
1H
11.01
9.09
8.24 8.23
7.75 7.73 7.50 7.49 7.48 7.47 7.47 7.43 7.26 6.83 6.75
5.30 5.23 5.21 5.20 5.19 5.18
4.25 4.22 4.19 4.17 4.16 4.01 4.00 3.99 3.98 3.96 3.94 3.92 3.78 3.76 3.75 3.72 2.57 2.56
1.64
1.26 1.24 1.22
Signal at 1.24 ppm is traces of EtOH (~3% by weight)
Signal at 5.30 ppm is traces of DCM
1.03 1.00 1.01 1.02 4.85 1.19 1.04 2.08 5.09 1.10 2.98 1.15
11.0 10.5 10.0 9.5 9.0 8.5 8.0 7.5 7.0 6.5 6.0 5.5 5.0 4.5 4.0 3.5 3.0 2.5 2.0 1.5 1.0
f1 (ppm)

SC11

LCMS Analysis Report

Compound ID: SC-11(HDBA0527-18-1)
Pump A : 0.1% formic acid in 100% water
Pump B : 0.1% formic acid in 100% acetonitrile
Total Flow : 0.3ml/min
Volume : 0.2ul
wave-length : 190-500nm

Gradient:	Time	A	B
	0.01	80%	20%
	2.50	0%	100%
	2.60	80%	20%

Column: Waters BEH C18 2.1x50mm 1.7um

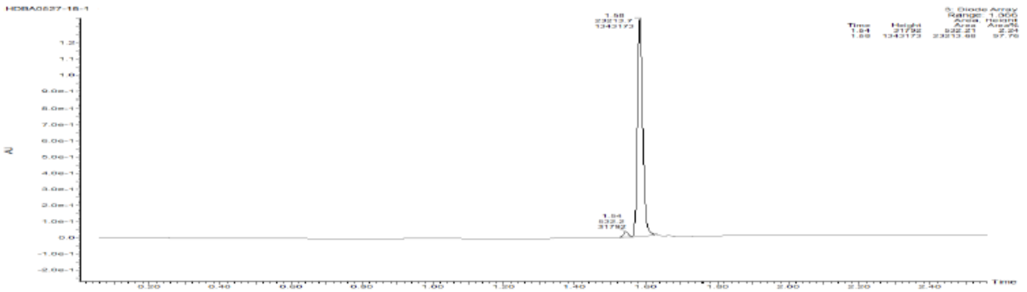

Peak number	Retention time(min)	Height(Au)	Area(Au*s)	Area%
1	1. 54	31792	532. 21	2. 24
2	1. 58	1343173	23213. 68	97. 76

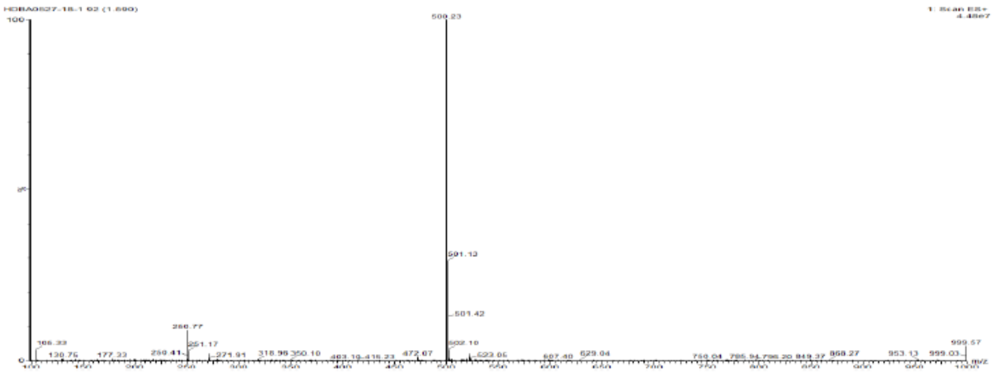

SC16

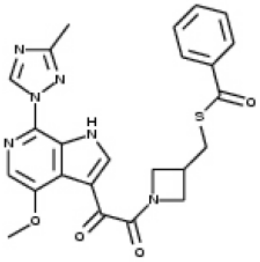

QP-130718-SC16_001 MW:490.5

==
Injection Date   : Fri, 27. Sep. 2013
Acq Operator   : 005654
Location   : P1-A-07
Inj. Vol.   : 2ul
Acq   Method   : E:\DATA\130927-WG2\WUXIAB01.M
Data Filename   : E:\DATA\130927-WG2\WG2IR1AG1.D
LCMS-W

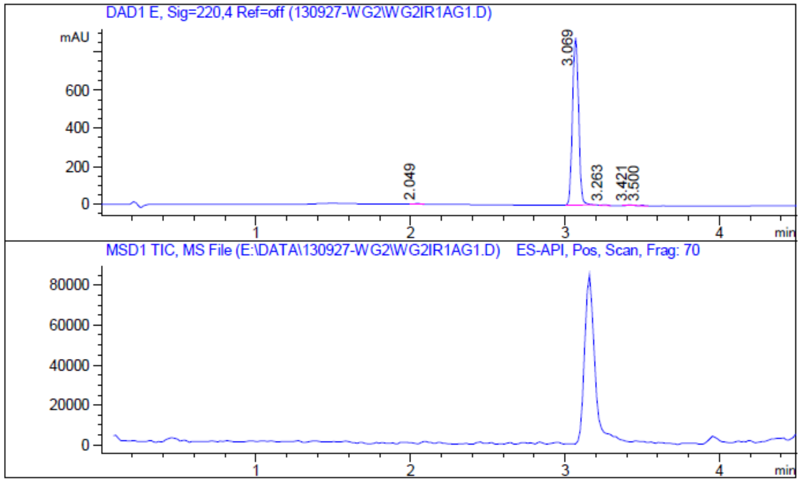

Report

Signal 1 : DAD1 E, Sig=220,4 Ref=off

Peak #	RT [min]	Area	Height	Height %	Width [min]	Area %
1	2.049	10.270	3.554	0.397	0.048	0.411
2	3.069	2456.402	881.848	98.407	0.046	98.321
3	3.263	7.080	2.563	0.286	0.046	0.283
4	3.421	16.383	5.132	0.573	0.053	0.656
5	3.500	8.220	3.022	0.337	0.045	0.329

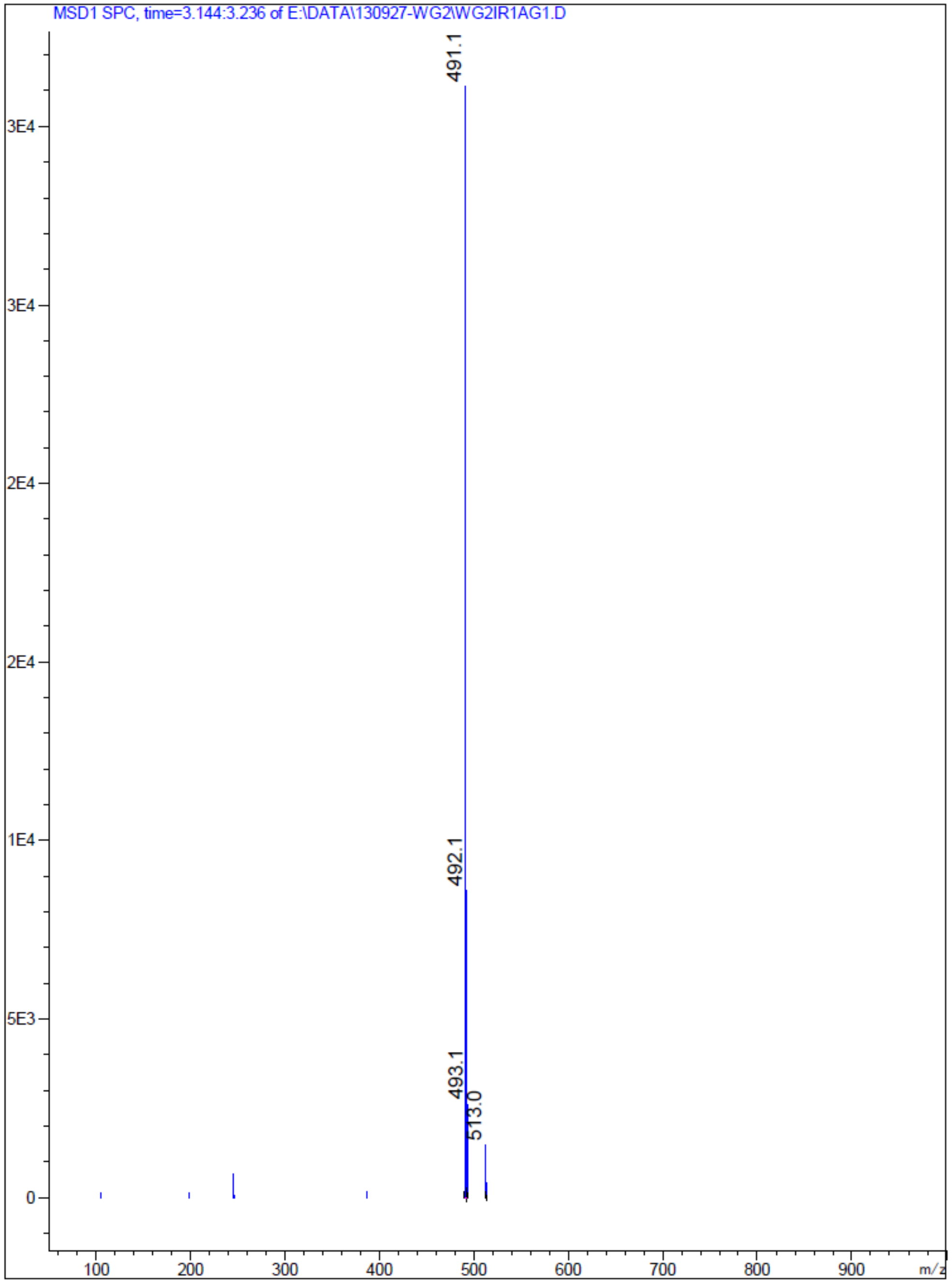
MSD1 SPC, time=3.144:3.236 of E:\DATA\130927-WG2\WG2IR1AG1.D
491.1
492.1
493.1
513.0
3E4
3E4
2E4
2E4
1E4
5E3
0
100
200
300
400
500
600
700
800
900
m/z
